# Differential Contribution of Malic Enzymes during Soybean and Castor Seeds Maturation

**DOI:** 10.1371/journal.pone.0158040

**Published:** 2016-06-27

**Authors:** Mariel Claudia Gerrard Wheeler, Cintia Lucía Arias, Silvana Righini, Mariana Beatriz Badia, Carlos Santiago Andreo, María Fabiana Drincovich, Mariana Saigo

**Affiliations:** Centro de Estudios Fotosintéticos y Bioquímicos (CEFOBI-CONICET), Universidad Nacional de Rosario (UNR), Rosario, Santa Fe, Argentina; Universidade Federal de Vicosa, BRAZIL

## Abstract

Malic enzymes (ME) catalyze the decarboxylation of malate generating pyruvate, CO_2_ and NADH or NADPH. In some organisms it has been established that ME is involved in lipids biosynthesis supplying carbon skeletons and reducing power. In this work we studied the MEs of soybean and castor, metabolically different oilseeds. The comparison of enzymatic activities, transcript profiles and organic acid contents suggest different metabolic strategies operating in soybean embryo and castor endosperm in order to generate precursors for lipid biosynthesis. In castor, the malate accumulation pattern agrees with a central role of this metabolite in the provision of carbon to plastids, where the biosynthesis of fatty acids occurs. In this regard, the genome of castor possesses a single gene encoding a putative plastidic NADP-ME, whose expression level is high when lipid deposition is active. On the other hand, NAD-ME showed an important contribution to the maturation of soybean embryos, perhaps driving the carbon relocation from mitochondria to plastids to support the fatty acids synthesis in the last stages of seed filling. These findings provide new insights into intermediary metabolism in oilseeds and provide new biotechnological targets to improve oil yields.

## Introduction

Seeds are the structural units that allow the propagation of higher plants and are usually of great economical interest because of the value of its organic composition. The final content of oil, proteins and starch in seeds varies with the species [1]. Soybean (*Glycine max*) seeds contain about 20% of triacylglycerols on average, while in castor (*Ricinus communis*) the storage lipids can account for almost 64% of the dry weight of the seed. The reserve storage occurs during the maturation stage, after the embryogenesis and prior to seed desiccation [2]. Besides the differences in the composition among the species, the seed maturation process has spatial variations regarding to reserve deposition. For example, in soybean most of the reserve accumulation occurs in the embryo; while in castor, the endosperm is the preferred tissue. Another difference is given by the role of light in the seed physiology; while castor seed is classified as not green [3], the photosynthesis could contribute to the generation of reserves in soybean seeds [4]. These oilseeds have important roles providing feedstock for human and animal diet and also to industrial processes. Soybean is one of the most versatile and important crop worldwide and constitutes the source of high quality oil with applications in food, textile, plastic and fuel industries [5]. Argentina, the world's largest exporter of soybean oil, harvested 55 million tons of soybeans in 2014, constituting the main income of foreign currency for the country. On the other hand, castor has great advantages as a fuel oil producing species. It was established that the castor oil is composed almost exclusively of triricinolein, which makes it very suitable for producing biodiesel since it does not require heat for the esterification [6]. Additionally, the castor plant can grow in semiarid climates and nutrient depleted soils, so it would not compete with the traditional oilseed crop species for the cultivable areas.

The generation of triacylglycerols in the seeds depends on the nutrients supplied from the autotrophic tissues of the plant. *De novo* fatty acid synthesis occurs in plastids and requires carbon skeletons (acetyl-CoA), energy (ATP) and reducing equivalents (NAD(P)H). In soybean and castor seeds, pyruvate and malate imported from the cytosol were proposed to be the main precursors for fatty acid synthesis [7]. In castor endosperm, the glycolytic conversion of glucose into phosphoenolpyruvate (PEP) followed by the action of pyruvate kinase or PEP carboxylase and malate dehydrogenase (MDH) result in cytosolic pyruvate or malate, respectively. Both metabolites can be imported into the plastids and transformed into acetyl-CoA, but malate has to be first converted to pyruvate by a malic enzyme (ME) before the pyruvate dehydrogenase (PDH) complex converts the pyruvate into acetyl-CoA [8]. In soybean embryos the same pathways could be operating; but besides sugars, amino acids should also be considered as carbon sources since they are readily available in this nitrogen-fixing plant. In this way, metabolic flux analyses have demonstrated that the carbon skeletons derived from the catabolism of amino acids can be converted to malate and pyruvate to sustain the fatty acid synthesis at least partially [4].

The malic enzyme (ME) catalyzes the oxidative decarboxylation of malate thus generating pyruvate, CO_2_ and a reduced cofactor, NADH or NADPH depending on the enzyme. In some non-plant organisms it has been established that ME is involved in the biosynthesis of lipids. Such is the example of *Mucor circinelloides*, a commercially used oleaginous fungus which shows a direct correlation between ME enzyme activity and lipid content [9]. Similarly, in mammals the cytosolic isoform of NADP-ME is involved in the fatty acids synthesis in liver and adipose tissue [10]. The comprehensive studies of the ME family from the model plants *Arabidopsis thaliana* and maize have shown multiple genes differing in expression patterns and catalytically distinctive protein products. NADP-ME isoforms are located in cytosol or plastids while NAD-ME are exclusively targeted to mitochondria [11–13]. This diversity can account for the great variety of functions that have been attributed to this enzyme in plants [14]. In this work we studied the NAD- and NADP-ME families of soybean and castor to examine if there is a special contribution of any isoform during seed maturation. The comparison of the family members on the basis of expression patterns, enzymatic activities and organic acids contents suggests differences in the roles of NAD- and NADP-MEs during the development of these oilseeds. Most significantly, NAD-ME seems to play an outstanding role in the maturation of soybean seeds, which prompted us to postulate an operating transport of citrate from the mitochondria as a mean to relocate carbon skeletons to support fatty acids synthesis in plastids.

## Materials and Methods

### Plant material

**Fig 1:**
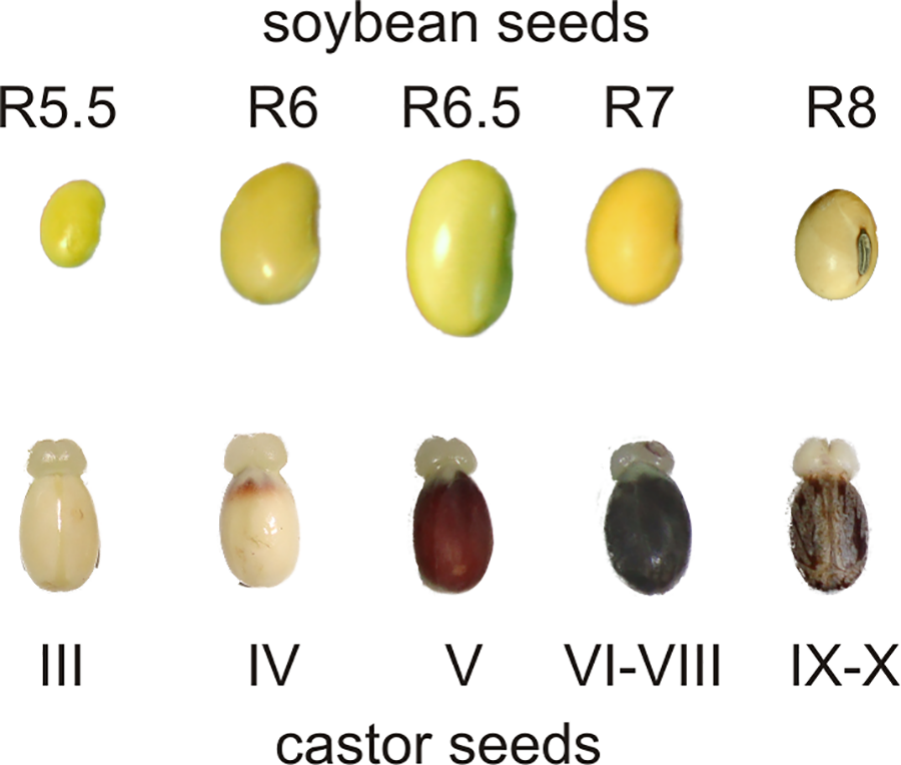
Maturing soybean and castor seeds collected for this work. The samples span the period of lipids biosynthesis already established in [15; 16].

Soybean (*Glycine max* Pioneer 94m80) and castor (*Ricinus communis* L. var. *Sanguineus*)were grown in a greenhouse- in soil pots weekly supplemented with 0.5X Hoagland solution under natural light supplemented with metal-halide lighting to provide a 16 hours light regime and temperatures between 20°C (night) and 30°C (day). The soybean seeds were harvested and classified by the following fresh weight ranges and colors: 75–100 mg (green), 200–300 mg (green), 400–500 mg (green-yellow), 400–300 mg (yellow) and 200–100 mg (dry, yellow), which correspond to the reproductive stages that span the filling phase R5.5, R6, R6.5 and R7 and the final desiccated stage R8, respectively (Fig 1) [15]. The seeds were dissected to separate the seed coat from the embryo, which was then stored at -80°C. Castor seeds were harvested and classified in maturation stages III, IV, V, VI-VII-VIII and IX-X, according to the morphological parameters described by [2] (Fig 1). By stage III, the seed has reached full length and fresh weight. Then, from stages IV to VIII the fresh and dry weights of the endosperm increase rapidly [2]. The seeds were dissected to separate the embryos and the seed coat from the endosperm, which was then stored at -80°C. In addition, samples of whole germinated soybean of 2, 4 and 7 days (G1, G2 and G3) and leaves, stems and roots (L, S and R) of soybean seedlings of 19 days were collected.

### Sequence and phylogenetic analyses

Protein sequences were retrieved from Phytozome 9.1 database (www.phytozome.net), using as query *A*. *thaliana* NADP-ME At5g11670 or NAD-ME At4g00570. The classification of each protein as NAD- or NADP-dependent, was further supported by the identity analysis derived from the ClustalW alignment of all the protein sequences (S1 Table). The prediction of the subcellular localization was performed with the TargetP tool. For phylogenetic analysis, we included all sequences present in each organism. The tree was inferred by neighbor joining method using MEGA 5.10 software. In order to evaluate the robustness of the tree structure, 100 replicates of bootstrap searches were performed.

### RNA extraction and transcripts quantification

Total RNA was isolated from 100 mg of each sample using the Trizol reagent (Life Technologies), further purified by a column-based method (PureLink, Life Technologies) and treated with RQ1 DNase (Promega) following the recommendations of the manufacturers. The concentration and purity of the preparations were measured spectrophotometrically and the integrity was assayed by agarose 2% (w/v) gel electrophoresis. Two micrograms of total RNA were reverse transcribed with 200 U of SuperScriptII (Life Technologies) using oligodT as primer.

Relative gene expression was determined by quantitative real-time PCR (qRT-PCR) on a Mx3000P system using the MxPro software version 4.10 (Stratagene). Specific primers of similar GC content and Tm were designed using the web-based tool Primer-BLAST (http://www.ncbi.nlm.nih.gov/tools/primer-blast) to allow for the amplification of 80–200 bp products. The PCR mix contained 1X SYBR Green I (Life Technologies), 0.2 mM dNTPs, 3 mM MgCl2, 0.25 μM of each primer, 0.025 U Platinum Taq DNA Polymerase and 1X buffer provided by the manufacturer (Life Technologies). Tenfold dilutions of the cDNAs synthesized as described above were used as templates. Thermal cycling parameters were as follows: initial denaturation at 94°C for 2 min, 46 cycles of 96°C (10 s) 56°C (15 s) 72°C (20 s) for amplification and 10 min at 72°C for the final elongation. The denaturation curve for each PCR product was determined by measuring the fluorescence with increasing temperature from 65 to 98°C. For both species an actin (ACT) gene was used as reference, Glyma04g39380 from soybean and Rco30206.m000761 from castor [6; 17]. Each RNA sample was run in triplicate and repeated in at least two independent biological samples. Relative gene expression was calculated using a modified version of the comparative 2^-ΔΔCT^ method, the efficiencies were calculated as described in [18]. The oligonucleotide primers pairs used are listed in S2 Table.

The primer pairs that failed to amplify any target in cDNAs samples were tested with genomic DNA to confirm their functionality. The genomic DNA from soybean seeds was isolated by CTAB precipitation as in [19].

### Protein extraction and enzyme activity measurements

Pools of soybean embryos or castor endosperms from 7–10 seeds were homogenized (1:2; w/ v) in mortars in 100 mM Tris-HCl (pH 7.5) containing 0.5% (w/v) Triton X-100, 10% (v/v) glycerol, 2 mM EDTA, 5 mM MgCl_2_, 10 mM β-mercaptoethanol and 0.5% (v/v) protease inhibitors cocktail (Halt Protease Inhibitor Cocktail, Thermo Scientific). Homogenates were centrifuged at 16,000 *g* for 15 min and the resulting supernatants were further clarified by centrifuging in the same conditions. Then, aliquots of 0.3 ml were desalted through 5 ml Sephadex G-50 spin columns. All procedures were performed at 0–4°C.

All activity assays were carried out at 30°C in a Jasco V-630 spectrophotometer in a final volume of 0.5 ml, following the production or consumption of NAD(P)H at 340 nm (ε_340nm_ = 6.22 mM^-1^ cm^-1^). One unit (U) is defined as the amount of enzyme that catalyzes the formation of 1 μmol of NAD(P)H min^-1^ under the specified conditions. For each enzymatic activity measurement, the reaction conditions were optimized considering substrate and cofactor concentrations, pH and linearity with time and protein extract concentration. Control assays without the addition of substrates, cofactors or protein extract were performed in each case. Each extract was measured by duplicate and repeated in two independent biological samples, each represented by 7–10 seeds collected from at least four plants.

NADP-ME activity was determined using a standard assay mix containing 50 mM Tris-HCl (pH 7.5), 10 mM MgCl_2_, 0.5 mM NADP, 10 mM L-malate and 5–10 μl of the protein sample. The reaction was started by the addition of L-malate [11]. NAD-ME activity was measured in a standard reaction mix containing 50 mM MES-NaOH (pH 6.6), 2 mM NAD, 10 mM L-malate, 10 mM MnCl_2_ and 10 units of porcine heart MDH. There was a rapid and small increase of the absorbance at 340 nm as the reaction catalyzed by the MDH reached the equilibrium. The NAD-ME reaction was started with the addition of 5–10 μl of the protein sample. With the assay system specified above, the subsequent steady increase of the absorbance after the addition of the sample was attributable to the decarboxylation of L-malate by the NAD-ME [20]. NAD-MDH activity was measured in a standard reaction mix contained 50 mM Tris-HCl (pH 7.2), 1 mM EDTA, 0.25 M sucrose, 0.02% (v/v) Triton X-100, 0.2 mM NADH and 2 mM oxalacetate. The reaction was started by the addition of 1–5 μl of the sample.

### Polar metabolites extraction and GC-MS analysis

All the procedures were carried out as described in [21]. Samples of soybean embryos or castor endosperms (100 mg FW) were homogenized in a mortar with liquid N_2_ and suspended in 1.4 ml of cold methanol. After the addition of 15 μg of ribitol, the extracts were incubated for 15 min at 70°C. Then, 0.75 ml of chloroform were added. Each tube was further incubated for 5 min at 37°C, diluted with 1.5 ml of distilled water and centrifuged at 2,200 *g* for 15 min. The polar phase was distributed in 400 μl aliquots and dried down under vacuum. Samples were derivatized by methoxyamination using a 20 mg/ml solution of methoxyamine hydrochloride in pyridine, and subsequent trimethylsilylation with MSTFA (N-Methyl-N-(trimethylsilyl) trifluoroacetamide). The derivate was injected into a VF-5ms column (Varian) using a GC-MS system Shimadzu QP 2010 plus. The running conditions are described in detail in [22]. Each sample obtained from 7–10 seeds was run in duplicate and repeated in three independent biological replicas. Signals were normalized to an internal standard molecule (ribitol). Furthermore,for the quantitative analyses, calibration curves were constructed using standard solutions of each metabolite tested. The chromatograms and mass spectra were evaluated using the AMDIS software.

### Statistical analysis

Data were tested using one way analysis of variance in all pairwise comparison type using the Holm-Sidak test (p < 0.05) with the SigmaPlot software.

## Results

### Soybean and castor NADP- and NAD-ME family members

**Fig 2:**
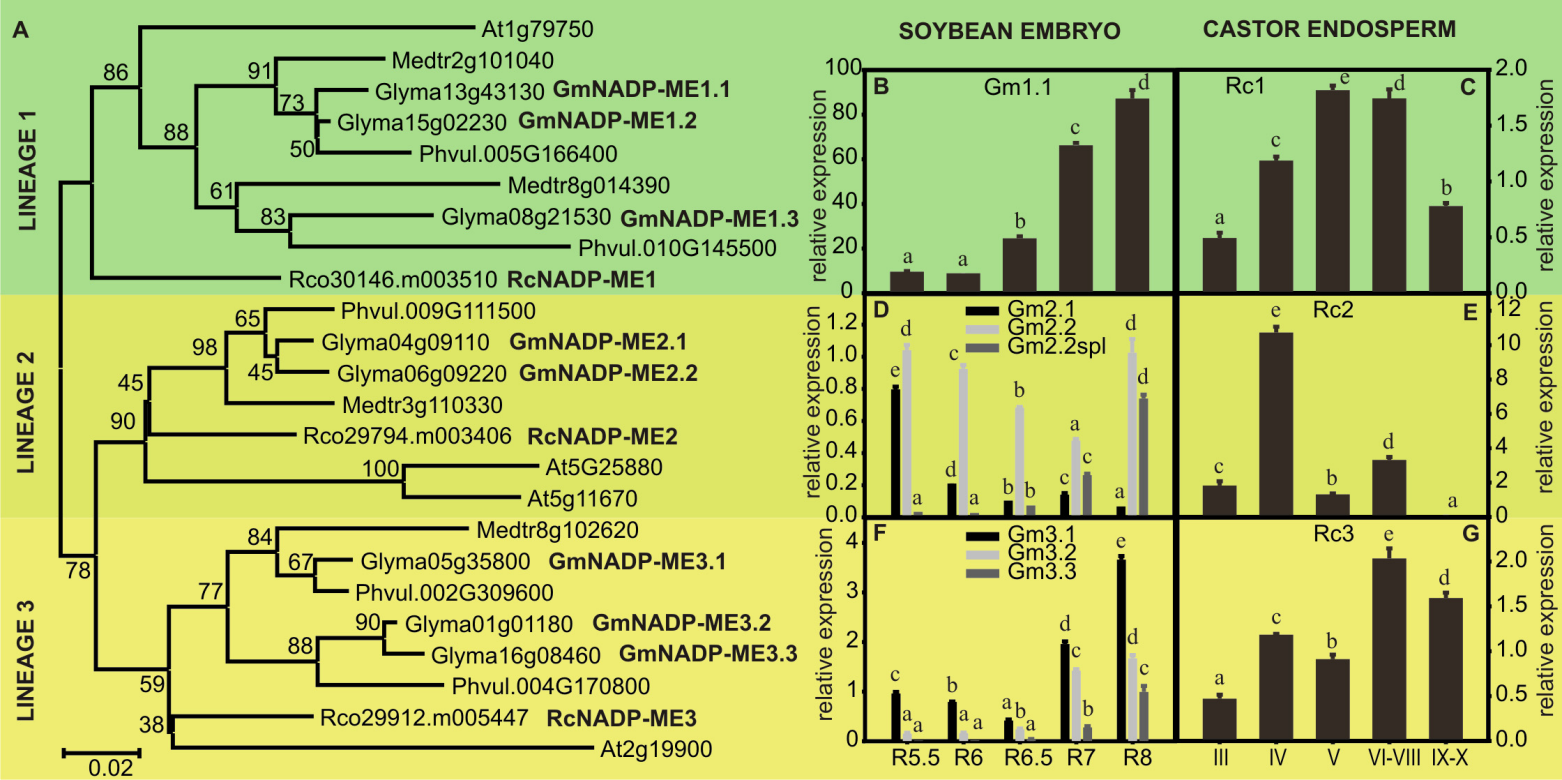
Phylogenetic tree of NADP-ME. (A). Protein sequences were aligned using Mega 5.10 and the phylogenetic tree was constructed by the neighbor joining method. The numbers indicate the statistical significance of each branch obtained by bootstrap analysis. Relative level of the transcripts of *NADP-ME* genes in maturing soybean embryos (B, D and F) and castor bean endosperms (C, E and G) determined by qRT-PCR. The functionality of primers for GmNADP-ME1.2 and 1.3 was verified using soybean genomic DNA. The primers for GmNADP-ME2.2 also anneals with the splicing version GmNADP-ME2.2spl. The actin genes Glyma04g39380 and Rco30206.m000761 were used as reference in soybean and castor, respectively. The values are the average of at least two independent experiments ± SD. For each transcript, values with the same letter are not significantly different (p < 0.05).

**Fig 3:**
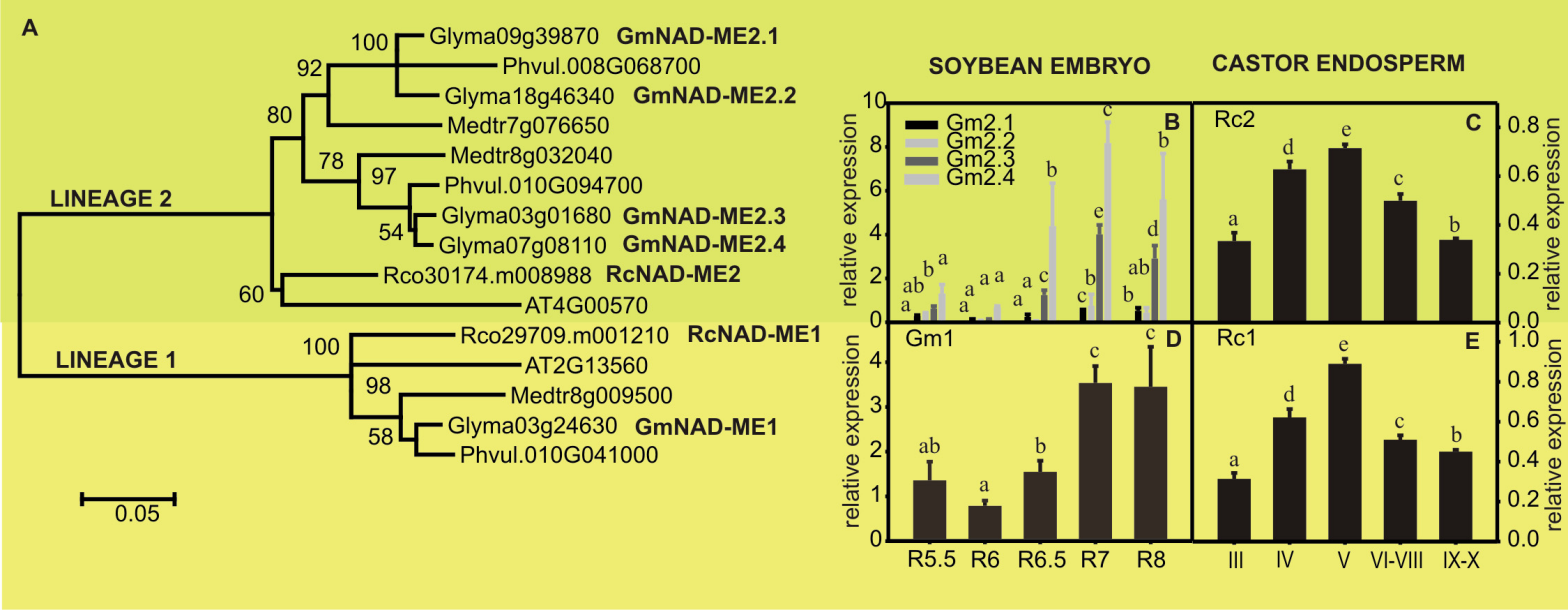
Phylogenetic tree of NAD-ME. (A). Protein sequences were aligned using Mega 5.10 and the phylogenetic tree was constructed by the neighbor joining method. The numbers indicate the statistical significance of each branch obtained by bootstrap analysis. Relative level of the transcripts of *NAD-ME* genes in maturing soybean embryos (B and D) and castor bean endosperms (C and E) determined by qRT-PCR. The actin genes Glyma04g39380 and Rco30206.m000761 were used as reference in soybean and castor, respectively. The values are the average of at least two independent experiments ± SD. For each transcript, values with the same letter are not significantly different (p < 0.05).

The search of ME genes in soybean and castor genomes was based on sequence similarity using *Arabidopsis thaliana* NADP-ME2 (At5g11670) or NAD-ME2 (At4g00570) as query. The high conservation in gene structure and protein length within plant malic enzyme families allowed us to identify truncated proteins resulting from the lack of conserved portions of exons/introns [11; 13]. Then, we continued the analyses with 14 putative malic enzyme sequences from soybean and 5 from castor. The high degree of conservation within NAD- and NADP- families allowed us to classify 9 and 3 sequences as putative NADP-MEs, and 5 and 2 sequences as putative NAD-MEs in soybean and castor, respectively (S1 Table). In order to distinguish orthologues, paralogues and recent gene duplications, we constructed two phylogenetic trees, one with all putative NADP-ME sequences here studied, and those from the model species *A*. *thaliana and two legumes (Medicago truncatula* and *Phaseolus vulgaris*) (Fig 2A) and another tree with all NAD-ME sequences from the same species (Fig 3A).

The NADP-ME tree topology shows three main lineages. Lineage 1 contains plastid-localized proteins and lineages 2 and 3 contain probable cytosolic enzymes, as deduced by *in silico* prediction analyses and previous targeting experiments (this work; [11; 23]). A tree that included sequences from a large number of eudicot species and some of the sequences analyzed in this work showed the same topology [24]. Among the legumes, *M*. *truncatula* and *P*. *vulgaris* display fewer NADP-ME genes than soybean in each lineage (Fig 2A), which agrees with their smaller genomes [25; 26].

Regarding NAD-ME tree, two lineages were obtained, lineage 1 defined by NAD-ME1 (At2g13560) and lineage 2 defined by NAD-ME2 (At4g00570) of *A*. *thaliana*, *both mitochondrial* proteins [12]. *In silico* prediction of soybean and castor NAD-MEs indicated high probabilities of mitochondrial locations. Lineage 1 contains one protein of each species but lineage 2 is more heterogeneous (Fig 3A). In this latter group, there is one castor protein and four of soybean, while *M*. *truncatula* and *P*. *vulgaris* have two proteins in this lineage.

### NAD(P)-malic enzyme transcripts profiles in maturing soybean and castor seeds

To analyze which of these genes are expressed in soybean and castor seeds, each transcript was quantified by qRT-PCR performed from RNA samples from all maturation stages (Fig 1). In castor endosperms the NADP-ME genes showed different expression patterns. The transcript Rco30146.m003510 (RcNADP-ME1) was the most abundant in the stages V and VI-VIII, while Rco29794.m003406 (RcNADP-ME2) was higher in stage IV and Rco29912.m005447 (RcNADP-ME3) accumulated mainly in the final stages (Fig 2C, 2E and 2G). In soybean, among lineage 1 genes, only Glyma13g43130 transcript (GmNADP-ME1.1) was detected in embryos, which increased from R6 stage onwards (Fig 2B). In lineage 2, Glyma04g09110 (GmNADP-ME2.1) decreased from R5.5 to R8 and Glyma06g09220 (GmNADP-ME2.2) also decreased from R5.5 to R7 but increased upon this stage (Fig 2D). A splicing variant of Glyma06g09220 that retains the 12^th^ intron of 87 bp (GmNADP-ME2.2spl) accumulated from R6.5 to R8 (Fig 2D). The *in vivo* role of the splicing is unknown and deserves further investigation. Regarding lineage 3 genes, Glyma05g35800, Glyma01g01180 and Glyma16g08460 (GmNADP-ME3.1, 3.2 and 3.3, respectively), all three showed maximal transcript levels at stages R7 and R8 (Fig 2F). Overall, the transcripts of lineage 3 have similar profiles in soybean and castor, while the expression patterns of lineages 1 and 2 genes are not conserved in both species.

In castor endosperms both NAD-ME transcripts (Rco29709.m001210, RcNAD-ME1 and Rco30174.m008988, RcNAD-ME2) reached maximal levels in stage V and then declined (Fig 3C and 3E). In soybean, Glyma03g24630 (GmNAD-ME1) transcript was more abundant in R7 and R8 (Fig 3D). Among cluster 2 transcripts, Glyma03g01680 (GmNAD-ME2.3) and Glyma07g08110 (GmNAD-ME2.4) were more abundant than Glyma09g39870 (GmNAD-ME2.1) and Glyma18g46340 (GmNAD-ME2.2) and accumulated from R6.5 to R8 (Fig 3B).

The high number of NADP-ME and NAD-ME genes in soybean could be indicating two contrasting situations: functional redundancy or functional differentiation. Then, we extended the expression analysis to other non-seed samples in order to determine if any of the genes is specifically expressed in seeds and if any of the non-expressed genes is actually active in other organs. The samples analyzed included germinating seeds and leaves, roots and stems from young plants. The putative transcripts GmNADP-ME1.2 and GmNADP-ME1.3, absent in soybean embryos, were not detected in neither of the new samples tested probably indicating inactive genes or an expression pattern very confined to temporal or spatial signals. Otherwise, GmNADP-ME2.2, GmNADP-ME2.2spl and GmNADP-ME3.3 were only found in maturing embryos raising the possibility that they are embryo specific transcripts. NAD-ME genes and GmNADP-ME3.1 were detected in similar levels in the other samples compared to embryo samples (S1 Fig). GmNADP-ME1.1 transcript was more abundant in embryos but GmNADP-ME2.1 and GmNADP-ME3.2 were detected at higher levels in non-embryo samples (S1 Fig). Overall, these observations show that genes of lineages 2 and 3 may have some functional specialization. Nevertheless, a broader analysis is needed to arrive to a certain conclusion about the physiological significance of each lineage in different organs.

### Activity profiles of malic enzymes in maturing soybean and castor seeds

**Fig 4:**
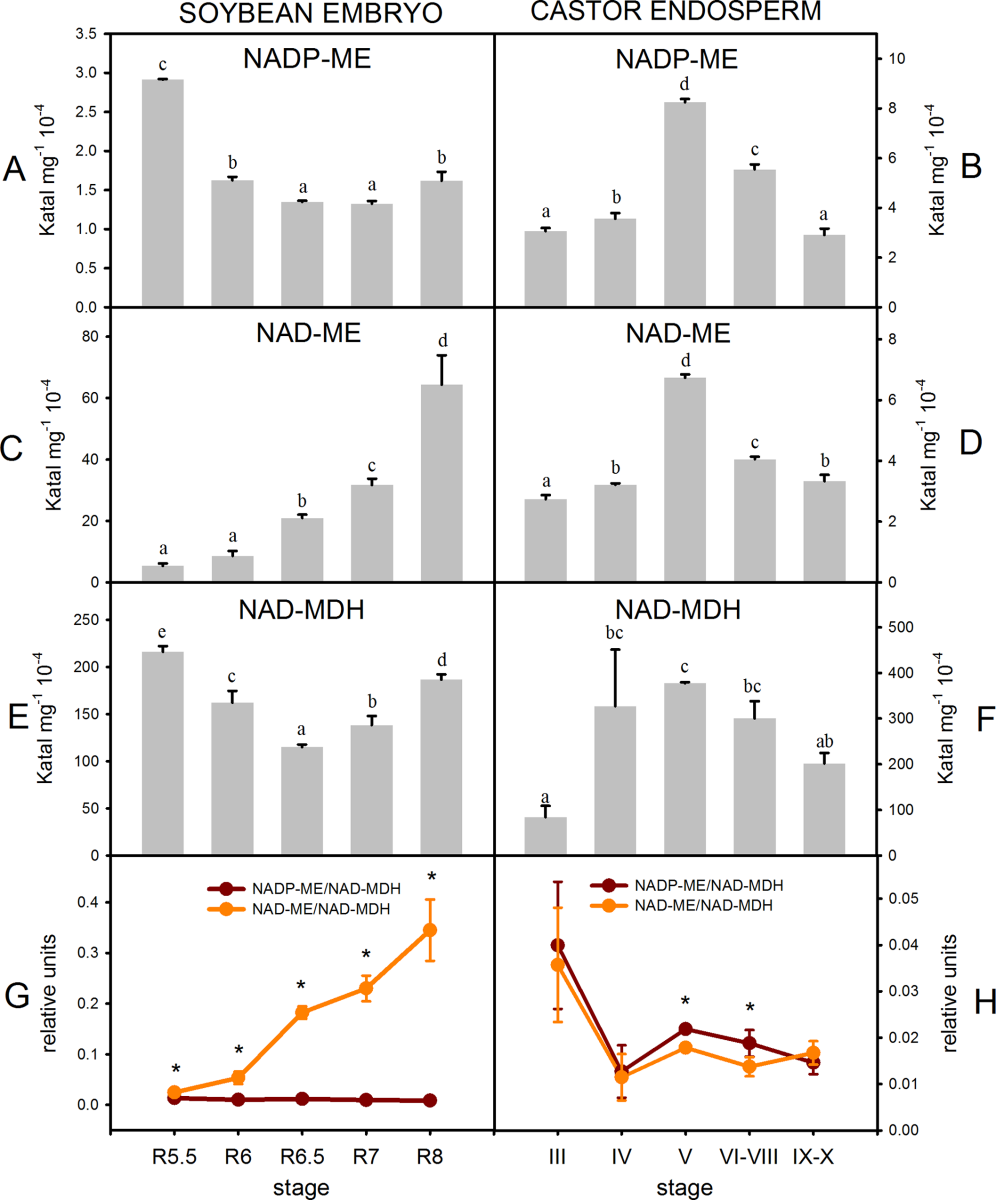
NADP-ME (A and B), NAD-ME (C and D) and NAD-MDH (E and F) activities in soybean embryos and castor endosperms. NAD(P)-ME/NAD-MDH ratios (G and H). One unit (U) is defined as the amount of enzyme that catalyzes the formation of 1 μmol of NAD(P)H min^-1^ under the specified conditions. FW: fresh weight. The values are the average of two technical duplicates from two independent samples ± SD. For each activity, values with the same letter are not significantly different (p < 0.05). In G and H, the asterisk denotes significant differences (p < 0.05) between the two ratios for each stage and for each species.

The NAD- and NADP-ME activities were assayed in soluble protein extracts from soybean embryos and castor endosperms. Both activities, expressed per gram of fresh weight (U.gFW^-1^), were comparable and followed similar profiles along castor seed maturation, reaching a maximum at stage V and then declining (Fig 4B and 4D). Since in stages VI-VIII and IX-X the endosperm accumulates higher contents of storage compounds (proteins and lipids) than in stage V, the decrease of U.gFW^-1^ levels can be partly explained by a dilution effect [27] besides a possible down-regulation of both activities. Then, it is not possible to infer the stages where the activities are up-regulated and most important for the metabolism. In the case of maturing soybean seeds, NAD- and NADP-ME activity profiles were completely dissimilar. While NADP-ME activity decreased from R5.5 to R6 and then maintained a constant level, NAD-ME increased during the maturation and showed up to 35 times higher activity than NADP-ME (Fig 4A and 4C). The activity of NAD- dependent malate dehydrogenase (NAD-MDH) was also assayed. This enzyme is abundant in plant cells and converts malate into oxaloacetate through a reversible reaction [28]. In both species, the total activity measured was higher compared to malic enzymes and showed similar profiles to NADP-ME activity (Fig 4E and 4F).

Since the fresh weight largely varies due to the accumulation of lipids and proteins and water loss in the stages analyzed, it is difficult to conclude about the fluctuations of an enzymatic activity between the different stages and to compare the same activity in both species. Then, to circumvent this dilution effect [27] we calculated the ratio between NAD- or NADP-ME activity to NAD-MDH activity for each sample to perform the comparative analysis (Fig 4G and 4H). In early stages of soybean seed maturation NAD-ME/MDH and NADP-ME/MDH were similar (Fig 4G). However, NAD-ME/MDH greatly increased as seed development proceeded due to a net increase of NAD-ME activity. It is worth noting that in R8 the activity of NAD-ME was 35% of NAD-MDH activity (Fig 4G), a value remarkably high since NAD-MDH activity is generally much higher than NAD-ME activity [12; 27; 28]. By contrast, the NADP-ME/MDH ratio in soybean remained low and nearly unchanged in the different stages analyzed (Fig 4G). On the other hand, in castor both ratios remained low and followed the same pattern (Fig 4H). These observations suggest a special contribution of NAD-ME in the maturing soybean seed metabolism and also highlight different metabolic functions of NAD-ME in soybean and castor seeds.

### Organic acid levels in maturing soybean and castor seeds

**Fig 5:**
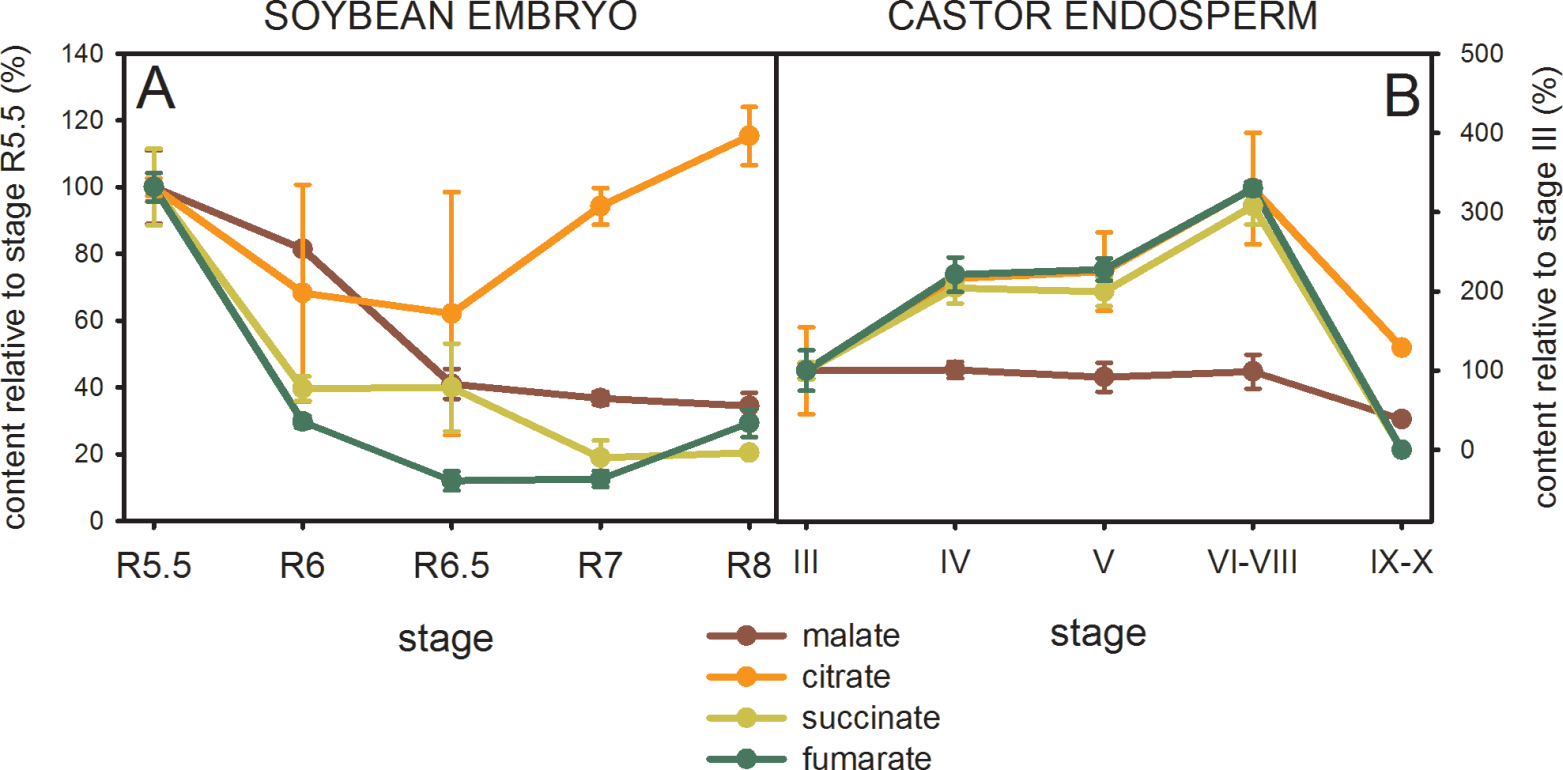
Organic acid content relative to stage R5.5 in soybean embryos (A) and stage III in castor endosperms (B) determined by GC-MS analysis. The peak areas were normalized according to the ones of the ribitol standard and the moles of each compound were determined using calibration curves. Each value was then relativized according to the fresh weight (FW) of each sample (S2 Fig). To facilitate the comparison between organic acids, the values were further relativized to the first stage analyzed in each species. The values are the average of three independent experiments ± SD.

We also analyzed the content of organic acids such as malate, citrate, succinate and fumarate in maturing soybean embryos and castor endosperms. Malate and citrate were highly abundant in both seeds, reaching levels similar to glucose and fructose; while succinate and fumarate concentrations were lower (S2 and S3 Figs). Since it is not possible to make direct comparisons of the metabolite levels due to the dilution effect previously mentioned, we compared the general profiles between metabolites in each oilseed (Fig 5). Succinate and fumarate followed similar accumulation patterns in each species. In castor, citrate also showed the same profile as succinate and fumarate but malate levels followed a different pattern (Fig 5B). In contrast, soybean malate accumulation pattern was more similar to succinate and fumarate patterns than to citrate, especially from R6.5 to R8 (Fig 5A). Overall, this analysis suggests that citrate and malate contribute differently to the metabolism in maturating soybean and castor seeds.

## Discussion

In this work we studied the complete set of *NAD-ME* and *NADP-ME* genes of soybean and castor. The number of *NADP-ME* and *NAD-ME* genes present in castor genome were similar to those found in other dicot C_3_ species such as *A*. *thaliana*, and tobacco (Figs 2A and 3A) [11; 29]. On the other hand, both gene families underwent a great diversification in legumes, evidenced as a higher number of isoforms present in soybean (Figs 2A and 3A). This can be explained by the events of whole-genome duplications that occurred during the evolution of legumes, one early in the legume stem clade and a more recent event specific of the genus *Glycine* [30]. Despite the different size of each gene family, in both oleaginous species we found encoded NADP-MEs of the three typical lineages [24] (Fig 2A] and NAD-MEs of the two lineages from dicots [14]. Except for GmNADP-ME1.2 and GmNADP-ME1.3, all transcripts were detected in samples of maturing soybean and castor seeds, although in different levels (Figs 2 and 3). The NADP- and NAD-ME activity profiles (Fig 4) point out that NAD-ME probably has a significant role in the metabolism of maturing seeds in soybean. The relative expression analysis shows that all the *NAD-ME* transcripts can contribute to the total NAD-ME activity. Then, the putative presence of five NAD-ME polypeptides in soybean could allow many possibilities of native assembly for this enzyme, which is capable of being assembled as homo- and heterodimers with differential kinetic and regulatory properties in Arabidopsis [31].

**Fig 6:**
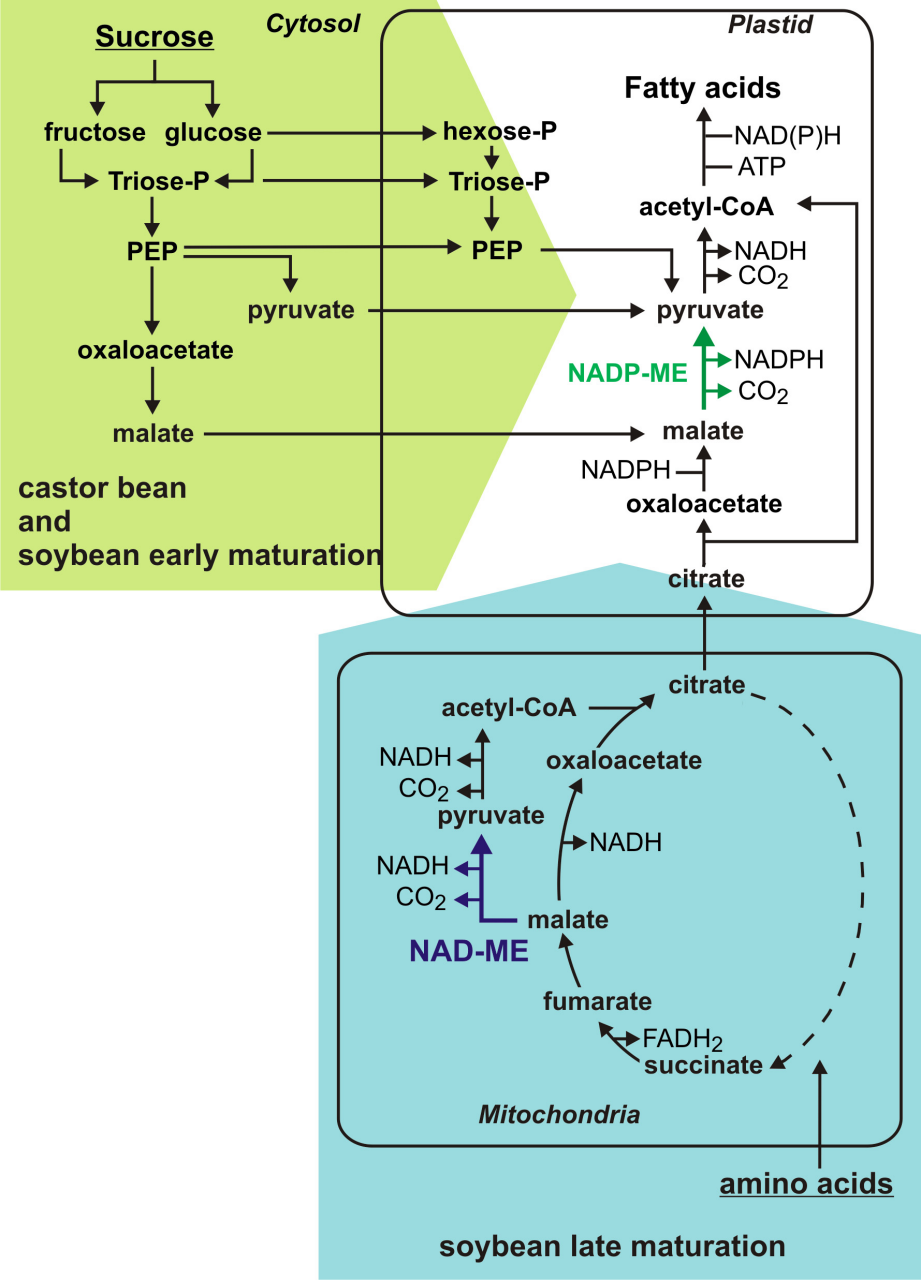
Proposed model for the generation of precursors for lipid biosynthesis in maturing soybean and castor bean. PEP, phosphoenolpyruvate; NADP-ME, NADP-dependent malic enzyme; NAD-ME, NAD-dependent malic enzyme.

The comparison of malic enzyme activities, transcript levels and intermediate compound contents suggests different metabolic strategies operating in soybean embryos and castor endosperms in order to generate precursors for reserve accumulation. Considering all above, we have developed a hypothetical model (Fig 6) in which the main differences would occur in the contribution of mitochondrial metabolism during the maturation stage, mainly represented by NAD-ME activity. In castor endosperm, the differential pattern exhibited by malate (Fig 5B) agrees with the central role of this organic acid as a precursor for the fatty acids synthesis in plastids previously proposed [8]. The synthesis of lipids in castor bean would rely mostly on cytosolic carbon derived from glycolysis which is partially imported into the plastids as malate (Fig 6). In this compartment, a plastidic NADP-ME would be essential to generate pyruvate, the immediate precursor of acetyl-CoA, and NADPH. In this sense, the genome of castor bean possesses a single gene encoding a putative plastidic NADP-ME (RcNADP-ME1), whose expression level is highest when the rate of lipid deposition is maximal (Fig 2C) [16].

In maturing soybean embryos, citrate level variations agree with a different role from those of malate, succinate and fumarate (Fig 5A). Accordingly, it was demonstrated by flux experiments that in early maturing soybean embryos the citrate synthesized in the mitochondria is incorporated in the plastids to provide carbon skeletons for fatty acid synthesis [32]. In this work, we found evidence that suggests that this pathway operates most significantly in latter stages during embryo maturation (Fig 6). At this point, the large increase of NAD-ME/NAD-MDH activity ratio (Fig 4G) would provide more pyruvate, and thus more acetyl-CoA, to promote the synthesis of citrate (Fig 6). These changes are reflected by the increase of citrate/malate ratio as maturation proceeds (S2 Fig). In embryo-culturing assays, the citrate export from the mitochondria to the plastids is enhanced by the decrease in the carbohydrates:amino acids ratio supply [32]. The carbon skeletons derived from the catabolism of amino acids enlarge the mitochondrial pool of organic acids which in turn are used for the synthesis of fatty acids. A similar situation could be found *in vivo*. As maturation proceeds, the carbohydrates supplied by photosynthetic organs would be diverted to other metabolism such as the synthesis of protective compounds that assist in the desiccation process [33]. In our study we could confirm that glucose-derived compounds such as galactinol and raffinose accumulate in soybean embryos from R6 stage (S4 Fig). Besides to overcome the shortage of glycolytic carbon products, the conversion of mitochondrial metabolites into plastidic reserve carbons could be a strategy to recycling carbon resources. Then, in this context soybean NAD-ME could play a central role in driving the relocation of the mitochondrial metabolic intermediates. While in the soybean early maturation, the glycolytic intermediates would be the main carbon source for fatty acid synthesis, in these later stages they would be replaced by TCA cycle intermediaries (Fig 6). Finally, in this model of carbon allocation from the mitochondria to the plastids, the plastidic NADP-ME would also function in the conversion of the citrate into acetyl-CoA (Fig 6). Although an increase in the total NADP-ME activity was not detected (Fig 4A), the transcript GmNADP-ME1.1, which would code for a plastidic isoform, was abundantly found in the last stages of maturation, even more than in other non-seed samples tested (Fig 2B and S1 Fig). Yet, this is not conclusive about a role in seed maturation since it may be indicating a role in the settings for the imminent germination [15].

The results presented in this work contribute to the understanding of carbon metabolism in oilseeds and provide a basis for improving reserve yields in these crops. Considering the different metabolic strategies operating in soybean embryo and castor endosperm, malic enzymes would have central roles in the supply of precursors for lipid biosynthesis and thus represent potential biotechnological tools.

## Supporting Information

S1 Fig **Relative level of the transcript of *NAD-ME* (B, D) and *NADP-ME* (A, C, E) genes in maturing soybean embryos (R5.5-R8), germinating soybean seeds (G1-G3) and leaves, stems and roots (L, S and R) of soybean seedlings determined by qRT-PCR.** The actin gene Glyma04g39380 was used as reference. The values are the average of at least two independent experiments ± SD. For each transcript, values with the same letter are not significantly different (p < 0.05).
(TIF)

S2 Fig Malate (A and B), citrate (C and D), succinate (E and F) and fumarate (G and H) content in soybean embryos and castor endosperms determined by GC-MS analysis. *non-detected. The peak areas were normalized to ribitol peak area and the moles of each compound were determined using calibration curves. Each value was relativized according to the fresh weight (FW) of each sample. The values are the average of three independent experiments ± SD. For each compound, values with the same letter are not significantly different (p < 0.05).
(TIF)

S3 Fig Glucose (A and B), fructose (C and D) and sucrose (E and F) contents in soybean embryos and castor bean endosperms determined by GC-MS analysis. The peak areas were normalized to ribitol peak area and the moles of each compound were determined using calibration curves. Each value was relativized according to the fresh weight (FW) of each sample. The values are the average of three independent experiments ± SD. For each compound, values with the same letter are not significantly different (p < 0.05).
(TIF)

S4 Fig Galactinol and raffinose levels during soybean embryos maturation. The peak areas were normalized to ribitol and refers to 1 g of fresh weight (FW) of each sample. The values are the average of three independent experiments ± SD. For each compound, values with the same letter are not significantly different (p < 0.05). *non-detected.
(TIF)

S1 Table Pairwise comparisons of amino acid sequences of MEs from Arabidopsis, castor and soybean. (PDF)

S2 Table qRT-PCR primers. (PDF)
