# Supplementary material for: Differential Contribution of Malic Enzymes during Soybean and Castor Seeds Maturation
Source: PLoS One. 2016 Jun 27;11(6):e0158040. doi: 10.1371/journal.pone.0158040 (PMC4922584; doi:10.1371/journal.pone.0158040)
Supplement: S1 Table — (PDF) [file pone.0158040.s005.pdf]

Table S1: Pairwise comparisons of amino acid sequences of MEs from Arabidopsis, castor and soybean. Percent Identity values are highlighted in different colors indicating low, medium or high values. Arabidopsis MEs sequences are: AtNAD-ME1 (AT2G13560), AtNAD-ME2 (AT4G00570), AtNADP-ME1 (AT2G19900), ANADP-ME2 (AT5G11670), AtNADP-ME3 (AT5G25880) and AtNADP-ME4(AT1G79750).

	AtNAD-ME1	AtNAD-ME2	AtNADP-ME1	AtNADP-ME2	AtNADP-ME3	AtNADP-ME4	Rc29709.m001210	Rc30174.m008988	Rc30146.m003510	Rc29912.m005447	Rc29794.m003406	Glyma07g08110	Glyma03g01680	Glyma09g39870	Glyma18g46340	Glyma03g24630	Glyma15g02230	Glyma05g35800	Glyma06g09220.1	Glyma06g09220.2	Glyma01g01180	Glyma16g08460	Glyma08g21530	Glyma04g09110	Glyma13g43130
AtNAD-ME1		61.0	41.0	40.3	40.1	37.4	82.2	64.0	38.8	40.8	40.7	62.9	62.2	62.2	62.2	82.8	38.2	39.2	40.9	38.4	40.1	38.5	39.5	41.5	37.7
AtNAD-ME2	61.0		38.4	37.9	39.8	37.4	62.8	81.7	37.4	39.4	39.3	81.1	79.3	77.7	78.0	63.8	37.9	37.1	38.0	36.4	38.4	37.1	38.6	38.4	37.6
AtNADP-ME1	41.0	38.4		76.3	75.2	76.1	41.7	39.9	79.5	83.0	79.7	40.1	38.7	39.1	39.1	41.8	78.0	81.4	79.4	79.4	81.6	81.8	76.4	79.9	78.0
AtNADP-ME2	40.3	37.9	76.3		91.0	75.7	39.8	39.1	79.6	79.6	80.6	39.0	37.8	37.2	37.2	39.1	76.5	78.7	80.3	80.3	77.7	77.6	75.3	81.1	76.2
AtNADP-ME3	40.1	39.8	75.2	91.0		75.3	39.5	39.1	77.9	80.3	80.6	39.8	38.3	37.9	37.9	39.1	75.7	77.6	79.6	79.6	77.0	77.6	74.8	80.4	75.7
AtNADP-ME4	37.4	37.4	76.1	75.7	75.3		37.2	38.5	77.2	80.9	80.1	37.8	38.9	36.9	37.0	37.9	77.2	73.1	78.1	74.4	78.0	75.6	80.1	78.1	76.5
Rc29709.m001210	82.2	62.8	41.7	39.8	39.5	37.2		65.5	38.7	40.1	40.5	63.9	63.1	63.7	63.8	87.0	37.4	38.5	40.1	38.0	39.9	38.5	39.1	40.1	36.9
Rc30174.m008988	64.0	81.7	39.9	39.1	39.1	38.5	65.5		38.7	39.9	41.2	86.4	85.3	84.2	85.3	67.1	38.9	38.5	38.4	36.9	39.1	38.2	39.8	39.3	38.5
Rc30146.m003510	38.8	37.4	79.5	79.6	77.9	77.2	38.7	38.7		86.5	84.0	38.6	38.0	37.9	37.8	38.1	79.7	78.2	83.4	79.5	83.8	80.7	83.5	83.8	80.7
Rc29912.m005447	40.8	39.4	83.0	79.6	80.3	80.9	40.1	39.9	86.5		83.8	39.8	38.3	38.1	38.1	39.9	82.1	87.5	83.0	82.7	87.0	88.0	80.3	84.2	82.1
Rc29794.m003406	40.7	39.3	79.7	80.6	80.6	80.1	40.5	41.2	84.0	83.8		40.5	39.4	39.1	39.0	40.0	80.8	82.8	85.9	85.9	82.0	81.8	78.2	85.9	80.8
Glyma07g08110	62.9	81.1	40.1	39.0	39.8	37.8	63.9	86.4	38.6	39.8	40.5		97.0	86.4	87.3	65.4	38.1	38.4	39.2	37.9	39.4	38.4	39.5	39.8	37.9
Glyma03g01680	62.2	79.3	38.7	37.8	38.3	38.9	63.1	85.3	38.0	38.3	39.4	97.0		84.3	85.5	64.8	38.0	39.4	38.0	37.6	38.3	37.8	38.3	38.5	37.8
Glyma09g39870	62.2	77.7	39.1	37.2	37.9	36.9	63.7	84.2	37.9	38.1	39.1	86.4	84.3		95.2	65.2	36.6	37.6	37.5	36.4	37.2	36.4	37.6	38.1	37.3
Glyma18g46340	62.2	78.0	39.1	37.2	37.9	37.0	63.8	85.3	37.8	38.1	39.0	87.3	85.5	95.2		65.0	36.7	37.7	37.4	36.3	37.1	36.7	37.8	37.9	36.5
Glyma03g24630	82.8	63.8	41.8	39.1	39.1	37.9	87.0	67.1	38.1	39.9	40.0	65.4	64.8	65.2	65.0		37.3	37.9	40.2	37.9	40.8	39.1	38.8	40.5	37.0
Glyma15g02230	38.2	37.9	78.0	76.5	75.7	77.2	37.4	38.9	79.7	82.1	80.8	38.1	38.0	36.6	36.7	37.3		77.1	81.2	77.4	79.7	77.4	87.4	81.5	97.4
Glyma05g35800	39.2	37.1	81.4	78.7	77.6	73.1	38.5	38.5	78.2	87.5	82.8	38.4	39.4	37.6	37.7	37.9	77.1		81.3	77.5	91.7	88.5	78.9	81.6	76.8
Glyma06g09220.1	40.9	38.0	79.4	80.3	79.6	78.1	40.1	38.4	83.4	83.0	85.9	39.2	38.0	37.5	37.4	40.2	81.2	81.3		100.0	81.0	80.8	79.1	98.0	81.3
Glyma06g09220.2	38.4	36.4	79.4	80.3	79.6	74.4	38.0	36.9	79.5	82.7	85.9	37.9	37.6	36.4	36.3	37.9	77.4	77.5	100.0		80.7	77.9	79.1	98.0	77.5
Glyma01g01180	40.1	38.4	81.6	77.7	77.0	78.0	39.9	39.1	83.8	87.0	82.0	39.4	38.3	37.2	37.1	40.8	79.7	91.7	81.0	80.7		97.6	78.4	81.3	79.5
Glyma16g08460	38.5	37.1	81.8	77.6	77.6	75.6	38.5	38.2	80.7	88.0	81.8	38.4	37.8	36.4	36.7	39.1	77.4	88.5	80.8	77.9	97.6		78.7	80.6	77.3
Glyma08g21530	39.5	38.6	76.4	75.3	74.8	80.1	39.1	39.8	83.5	80.3	78.2	39.5	38.3	37.6	37.8	38.8	87.4	78.9	79.1	79.1	78.4	78.7		79.4	87.1
Glyma04g09110	41.5	38.4	79.9	81.1	80.4	78.1	40.1	39.3	83.8	84.2	85.9	39.8	38.5	38.1	37.9	40.5	81.5	81.6	98.0	98.0	81.3	80.6	79.4		81.1
Glyma13g43130	37.7	37.6	78.0	76.2	75.7	76.5	36.9	38.5	80.7	82.1	80.8	37.9	37.8	37.3	36.5	37.0	97.4	76.8	81.3	77.5	79.5	77.3	87.1	81.1	

<42%   42%<x<72%   >72%
