# Supplementary material for: Differential Contribution of Malic Enzymes during Soybean and Castor Seeds Maturation
Source: PLoS One. 2016 Jun 27;11(6):e0158040. doi: 10.1371/journal.pone.0158040 (PMC4922584; doi:10.1371/journal.pone.0158040)
Supplement: S2 Table — (PDF) [file pone.0158040.s006.pdf]

Table S2: qRT-PCR primers.

Protein[1]	Transcript code[2]	Forward primer	Reverse primer	Product lenght (bp)
GmNADP-ME1.1	Glyma13g43130	TAGGCGCTTTTTTCTTTTTCATTTAT	TCCAAGTACCACTTCACTACCACC	155
GmNADP-ME1.2	Glyma15g02230	ATGTGTGTTTGTGTCAGTCCTTGG	TGCTGGATCATTTTCTTCACCTG	171
GmNADP-ME1.3	Glyma08g21530	CCACTAGCCTTCCTCAACCAAAA	TTGGGCAGAAAAGTAAGAAAAAACA	157
GmNADP-ME2.1	Glyma04g09110	AGCTTTGTGGTATAAGGCATTG	AAGCTCATGCTACTACAAAAATCAT	147
GmNADP-ME2.2[3]	Glyma06g09220.1.1	CAGAAAGATATCAGCCCACATTGC	TGGGCTGTACATGCAACTTTCTG	122
GmNADP-ME2.2spl	Glyma06g09220.1.2	GTAAAAATGCCGGGCTGGTTCTA	GATTGCCTTGACAGCATCCAGAA	192
GmNADP-ME3.1	Glyma05g35800	GGCTCTAACAAGGATGTGTTTCG	CAAAGTCAGCCAGGGCAAATG	196
GmNADP-ME3.2	Glyma01g01180	TTGTTATGGGGCAAATCACTGTTAG	TGCTTCACCC AAACACGCTA ATG	158
GmNADP-ME3.3	Glyma16g08460	TGATGCCATTGAATTTTATCAAGGAGG	CCATATTTCTGGCAAGCCTCACC	155
GmNAD-ME1	Glyma03g24630.1	CTCGGGAGCTGCAGAAACTA	CGAACCAAACATGCATCGAGA	154
GmNAD-ME2.1	Glyma09g39870.1	ACACTTCTGTCAGGTGCTCG	CTGCATGTACAACAGCAGCC	169
GmNAD-ME2.2	Glyma18g46340.1	TGATATACAGGGCACGGCTG	AGCCATGCTGAGAACACCAA	145
GmNAD-ME2.3	Glyma03g01680.1	CTCGATGGCCCAAGGCTATT	CAAGTGCAACACCAGCAGTG	132
GmNAD-ME2.4	Glyma07g08110.1	GGTGTTTGCAAGTGGAAGCC	TCCTCGGCCATGTATGAAGC	192
GmACT2/7	Glyma04g39380.1	GGAATTCACGAGACCACCTACAAC	GCTAGGAGCAAGTGCAGTGATTTC	153
RcNADP-ME1	Rco30146.m003510	AACATGCCAGTGGAAGAGAC	TCCCACTCCTGATGTTCCAA	192
RcNADP-ME2	Rco29794.m003406	GATATTCAGGGTACAGCATCCG	TGATTAGACAAGGTTCCGCC	80
RcNADP-ME3	Rco29912.m005447	AACCCATCTCGTCTTCAACG	GTGTGATCACTCAGGGAACC	105
RcNAD-ME1	Rco29709.m001210	TCAAGGATTAAGGGAAGGTGC	TTGCTGGTCTAGTTGAGGTTG	146
RcNAD-ME2	Rco30174.m008988	GAACTGTAAGAGCCCAAGGAC	AGCCATATTCAGGACACCAAG	101
RcACT	Rco30206.m000761	GTGTCAAGGTGTCATGTGAAC	AGAGATGGAAGGCTATTTGGTAC	102

[1] this work. [2] Schmutz et al., 2010 and Castor Bean Genome Database (http://castorbean.jcvi.org). [3] The primers for the transcript of GmNADP-ME2.2 also anneals with the transcript of GmNADP-ME2.2spl.
